# The study of antimicrobial, anti-cancer, anti-inflammatory and α-glucosidase inhibitory activities of Nigronapthaphenyl, isolated from an extract of *Nigrospora sphaerica*

**DOI:** 10.1080/21501203.2019.1620892

**Published:** 2019-05-24

**Authors:** Kushan M. Ukwatta, Jennifer L. Lawrence, C. D. Wijayarathna

**Affiliations:** a Department of Chemistry, University of Colombo, Colombo, Sri Lanka; b Department of Chemistry and Earth, Ocean and Atmospheric Science, University of British Columbia (UBC), Vancouver, Canada; c AMEDA Diagnostics, Krenngasse, Austria

**Keywords:** Endophytic fungi, Bruguiera gymnorrhyza, Nigrospora sphaerica, Nigronapthaphenyl

## Abstract

A new compound, nigronapthaphenyl, was extracted from the endophytic fungus *Nigrospora sphaerica* isolated from a mangrove plant *Bruguiera gymnorrhyza*. The structure of the compound was elucidated by analysis of 1D and 2D NMR spectra and mass spectrometric data. It was tested *in vitro* for its antimicrobial activity, cytotoxicity, anti-inflammatory activity and for its ability to inhibit α-glucosidase. Nigronapthaphenyl showed antibacterial activities against *Bacillus subtilis* TISTR 088 and *Bacillus cereus* TISTR 688 with MIC values of 4 and 2 μg/mL respectively. Cytotoxicity against colon cancer cell line HCT 116 was found to be an IC_50_ value of 9.62 ± 0.5 μM . This further showed potential anti-inflammatory activity amounting to an IC_50_ of 6.2 ± 0.5 μM and also α-glucosidase inhibitory activity, with an IC_50_ value of 6.9 ± 0.5 μM.

## Introduction

There is a need to search for new antimicrobial agents because infectious diseases still pose a global problem due to emergence of drug-resistant pathogens. (Espada et al. 1997; Zhang et al. 2000; Selim et al. 2012) As a result of diverse cancer types, levels of worldwide mortality rates have elevated. Therefore, synthesis of novel anti-cancer drugs has become a crucial requirement. (Kharwar 2011) Different microorganisms inhabit fauna and flora on surface or within cells, giving beneficial as well as non-beneficial effects to the host. Those microorganisms that inhabit healthy plant tissues without causing any apparent harm to the host are known as endophytes. (Huang et al. 2001) Endophytic fungi have been found in majority of plant families examined to date. They are viewed as an outstanding source of secondary metabolites and bioactive natural products such as alkaloids, terpenoids, steroids, quinones, isocoumarin derivatives, flavanoids, phenols, phenolic acids, and peptides. (Amarasinghe and Balasubramaniam 1992) Some endophytic fungi have the ability to produce the same or similar plant derived bioactive compounds as those originated from their host plants, such as paclitaxel, podophyllotoxin, camptothecine, vinblastine, hypericin, and diosgenin. (Clardy et al. 2009)

Antimicrobial agents are substances produced by various species of microorganisms, namely as, bacteria, fungi and actinomycetes to suppress the growth of other microorganisms and to eliminate them. These drugs have greatly reduced illnesses and deaths from infectious diseases such as bacterial meningitis, neurosyphilis, endocarditis, skin infections *etc*. (Berdy 2005) However, due to considerable development of antimicrobial resistance by certain pathogens, these antibiotics have rendered to be largely ineffective. Examples are methicilin resistant *Staphylococcus aureus* (MRSA), vancomycin resistant *Enterococci* (VRE), tuberculosis strains resistant to isoniazid and rifampicin (multi-drug resistance – MDR-TB) and fluoroquinolone resistant TB (XDR-TB). (Reichenbach and Hofle 2002) Infections caused by resistant microorganisms often fail to respond to conventional treatments, resulting prolonged illnesses and greater risk of deaths. In addition, life threatening systemic fungal infections which were rare in humans have now become more prevalent. (Xu et al. 2011) Hence there is an urgent need to develop more effective types of antibiotics to deal with these lethal, drug-resistant infections.

A vast majority of antimicrobial agents currently in clinical use has been isolated from soil-microbes. However, due to high rate of rediscovery and increased antimicrobial resistivity to available pathogens, developing clinically useful antibiotics from soil microbes have become unproductive and uneconomical. Therefore, it is a necessity to search for alternative sources. (Romagnoli et al. 2006, 2008; Weber et al. 2011) One of the major alternatives is the endophytes, which are widespread and apparently residing in all major plant species in the world. Endophytic fungi possess vastly untapped resources, which can be utilized for this purpose, as they are known to biosynthesize secondary metabolites of diverse structures and biological activities. (Park et al. 2003) Exploiting endophytic fungi from plants is environmentally friendly, since only a minute part (leaf, flower, bark and fruit) of the plant is used for investigation. Once the cultures are preserved they are renewable and have several advantages such as being able to be stored perpetually and manipulated genetically to optimize their utility. (Liu et al. 2010)

On this attempt to identify drug leads from compounds isolated from endophytic fungi, our research team successfully obtained many compounds from endophytic fungi resident in terrestrial and aquatic plants, such as Mycoleptodiscin B isolated from the endophytic fungus *Mycoleptodiscus sp*. (Dissanayake et al. 2016), chaetoglobosin A and C, produced by the endophytic fungus *Chaetomium globosum, (*Ranga Dissanayake et al. 2016) equisetin, from endophytic *Fusarium sp. (*Ratnaweera et al. 2015a) and solanioic acid, an antibacterial degraded steroid produced in culture by the fungus *Rhizoctonia solani. (*Ratnaweera et al. 2015b)

Although relatively higher number of studies has been conducted to isolate biologically active compounds of endophytes from terrestrial plants, mangrove plants and their associated endophytic fungi have not been investigated, in spite of their rich availability of bioactive molecules, and only a limited antimicrobial screening of this chemodiverse source has been reported. (Ratnaweera et al. 2016) Mangrove forests are fascinating and complex ecosystems. (Romagnoli et al. 2007) They serve coastal populations worldwide by protecting shorelines from storm surge and erosion, through filtration and remediation of terrestrial runoff, and as nurseries for important fisheries, among other useful roles. (Mathew et al. 2007)

## Materials and methods

### Collection of plant material

Fresh healthy leaves, stem, flowers, bark and fruits of *Bruguiera gymnorrhyza* were collected from Attaragoda Wetland (Latitude: 6° 1′ 60 N, Longitude: 80° 15′ 0 E); situated in the south coastal region about 3 km into the country from the main city of Galle, Sri Lanka. The collection was made in November 2013, transported to the laboratory in a tightly sealed Zip bag and stored at room temperature (30°C) under humid conditions. All respective parts of the tree were used for isolation of endophytic fungi within 16 hours.

### Isolation of endophytic fungi

The plant materials were surface sterilized with 70% ethanol and 5% sodium hypochlorite as in Radji et al. (2011). (Radji et al. 2011) Squares of about 0.5 cm^2^ obtained from the surface sterilized plant parts were placed on potato dextrose agar (PDA) (Himedia) medium in Petri dishes. The dishes were incubated at room temperature (30°C) until the emergence of hyphae.

After 6 days, edges of the small growth rods (mycelium growing) of the plant materials were cut and transferred onto fresh PDA dishes. Serial sub culturing was done until pure cultures were obtained. The isolated pure fungus was stored as PDA slants in glycerol and in distilled water at −10°C for future cultivation. (Radji et al. 2011)

### Identification of the endophytic fungus

The isolated endophytic fungus responsible for the secretion of the major compound was done by 16S rRNA analysis. Fungal DNA was extracted in the laboratory using the protocol of Kariyawasam et al. (2012). (Kariyawasam et al. 2012) The extracted DNA was subjected to the polymerase chain reaction (PCR) using universal primers ITS1 and LR3. Amplified DNA was subjected to DNA sequencing and obtained DNA sequence was compared with already existing DNA sequences in NCBI GenBank*. The acquired gene sequence was submitted to the NCBI GenBank database and an accession number was obtained. http://www.ncbi.nlm.nih.gov.blast

### Large scale extraction and screening for antimicrobial activities

The isolated endophytic fungus was grown on PDA medium using 600 Petri dishes (100 × 20 mm) for 25 days at room temperature. Each dish had 14 mL of PDA. At the end of the incubation period, the medium together with the fungal mycelium in all dishes was cut into small pieces and immersed in analytical grade ethyl acetate (EtOAc) 5000 mL (1000 mL conical flasks ×5) for 48 hours and filtered through glass wool. This extraction process was repeated twice. The filtrates were combined and ethyl acetate was evaporated and the extract was brought to dryness under reduced pressure at room temperature using a rotary evaporator (BUCHI-R-200).

The crude extracts of the fungus were tested for antimicrobial activities against two Gram-positive bacteria, *Bacillus subtilis* (UBC 344), methicillin-resistant *Staphylococcus aureus* (MRSA, ATCC 33,591), two pathogenic Gram-negative bacteria, *Escherichia coli* (UBC 8161) and *Pseudomonas aeruginosa* (ATCC 27,853) and a pathogenic fungus *Candida albicans* (ATCC 90,028) at 50 μg/disc using the agar disc diffusion method (National Committee for Clinical Laboratory Standards 2003). (National Committee for Clinical Laboratory Standards (NCCLS) 2002)

### Fractionation, isolation and structure elucidation of the bioactive component

To isolate the principal bioactive component(s) from the complex mixture of the fungal extract, an advanced series of bioassay-guided purification steps were performed. The crude extract (900 mg) was first subjected to solvent/solvent partitioning between hexane and MeOH/H_2_O, 9:1 (1:1 300 mL of each) and left to sit still for 18 hours; after the separation of the hexane layer, the polarity of the aqueous layer was increased to MeOH/H_2_O, 1:1 by addition of H_2_O and extracted with CHCl_3_ (300 mL × 2 times). The CHCl_3_ layer was separated and the aqueous layer was concentrated at reduced pressure and was partitioned between H_2_O and EtOAc (200 mL × 3 times). All four fractions were tested for bioactivity and continued with the combined CHCl_3_ and EtOAc soluble fractions which had retained bioactivity. Next, the combined fraction (dry weight – 110 mg) was eluted through a Sephadex LH-20 size exclusion chromatography (2.5 × 175-cm column) with MeOH/CHCl_3_; 7:3 as the eluting solvent. Resulting fractions were combined according to the thin layer chromatography (TLC) profiles and the combinations were tested for antibacterial and antifungal activity. The most active fraction (dry weight – 22 mg) was run on a normal phase silica (2.5 × 60 cm) column using gradient elution (10% to 60% MeOH: CH_2_Cl_2_). The resulting active fraction (dry weight −10.2 mg) was then run on Waters Sep pak C18 (2 g) reversed phase cartridge (60%:40% H_2_0: MeOH) to further purify the active component. Finally the active fraction (3.9 mg) was purified by C18 reversed-phase high performance liquid chromatography (HPLC) using a CSC-Inertisil 150A/ODS2, 5 μm 25 × 0.94 cm column with 1:4 MeCN/H_2_O as eluting solvent with a flow rate of 2 mL.min^−1^ to yield 2.6 mg of the major compound.

The structure elucidation of the isolated compound was done using nuclear magnetic resonance (NMR), low resolution and high resolution mass spectral data. The ^1^H, ^13^C and 2D NMR data was obtained by a Bruker AV-600 spectrometer with a 5 mm CPTCI cryoprobe, while low and high resolution masses were recorded on a Bruker-Hewlett Packard 1100 Esquire–LC system and MAT 95 XL mass spectrometers respectively.

### Antibacterial activities of the isolated pure compound

The pure compound was tested for antimicrobial activity against three Gram-positive bacteria, *B. subtilis* (UBC 344), *S. aureus* (ATCC 43,300) and methicillin resistant *S. aureus* (MRSA, ATCC 33,591), two Gram-negative bacteria, *E. coli* (UBC 8161), *P. aeruginosa* (ATCC 27,853) and the pathogenic fungus *C. albicans* (ATCC 90,028) using disc diffusion assay. (Houghton 2000) The minimum inhibitory concentrations’ (MIC) end point was taken as the lowest concentration with more than 95% growth inhibition. The MICs were determined using broth micro-dilution method according to National Committee for Clinical Laboratory Standards (NCCLS, 2002) with modification using Mueller Hinton broth as the medium. Optical density of the microbial growth was determined (at 600 nm) using a DTX 880 (Beckman Coulter Inc.) plate reader. The commercially available antimicrobial agents polymyxin B for *B. subtilis, E. coli* and *P. aeruginosa*, rifamycin for *S. aureus* and MRSA and amphotericin for *C. albicans* were used as positive controls (Concentration series used as 8.0–0.004 μg mL^−1^, by utilizing commercially available reagent series from Roche Diagnostics – CAT 12,457).

### Screening for anti-inflammatory activity

Cell-based assay for THP-1 (human monocytic cell line derived from an acute monocytic leukemia patient) cytokine-release assay was conducted to quantify the anti-inflammatory assay. (Paige et al. 2011) The cell line medium was supplemented with 2 mm l-glutamine, 100 U of penicillin per mL, 100 mg of streptomycin per mL with 25 mm 4-(2-hydroxyethyl)-1-piperazineethanesulfonic acid (HEPES), and 10% fetal bovine serum (FBS) to affiliate initial cell adherence. Induction of cell differentiation was obtained with 100 nm phorbol-12-myristate-13-acetate (PMA) in 24 hours. After incubation, non-adherent cells were removed by aspiration, and the adherent cells were washed with Roswell park memorial institute medium (RPMI) three times. For cell stimulation, the cells were further incubated separately with lipopolysaccharides (LPS) for 24 hours in fresh complete medium with 10% FBS. After cell plating, the test compounds, 0.5% dimethyl sulfoxide (DMSO) were added to each well, and the plate was incubated for 30 min at 37.8 °C. Finally, 20 mL (10 mg/mL) of LPS per well were added, to obtain a final concentration of 1 mg/mL.

Blood was withdrawn from human volunteers and aliquoted to a 96 well plate (50 µL each). Immunomodulator was added (10 µL) and incubated at 37 °C, maintaining a 5% O_2_ concentration for 15 minutes before adding 10 µL (final concentration 10^^^5 cells/mL) of the *E. coli* inoculate. They were then incubated for 7 hours, followed by the addition of 100 µL of phosphate buffered saline (PBS). After mixing, the plate was centrifuged at 1200 rpm for 5 minutes, and the supernatant was obtained and used for IL6 analysis to check the inhibition percentage of the inflammation. (Paige et al. 2011)

### Detection of secreted cytokines

Human tumor necrosis factor alpha (TNF-α) and interleukin 6 (IL-6) were quantified by an enzyme-linked immunosorbent assay (ELISA) as described in Paige et al. (2011) Briefly, 96-well ELISA plates (Maxisorp; Nunc, Naperville, Ill.) were coated with an antihuman TNF-α/IL-6 monoclonal antibody in a coating buffer (carbonate/bicarbonate buffer, pH 9.6), followed by overnight incubation at 48 °C. The wells were blocked for 2 h, at room temperature with 10% FBS prepared in assay buffer. Biotinylated antihuman TNF-α/IL-6 polyclonal antibody was added, followed by avidin-horseradish peroxidase conjugate, which used tetramethylbenzidine as the substrate. The reaction was stopped by the addition of 2M H_2_SO_4_, and optical density was recorded in a Saphiremicroplate reader (Tecan) at 490 to 600 nm.

### Cytotoxic assay

HCT116 colon cancer cells (1 × 10 (Kharwar 2011) cells/well) were cultured in a 96-well plate and allowed to adhere for 24 h at 37°C. The cells were treated with compounds (10 μM from 1 μg/mL solution) in Dulbecco’s modified eagle medium (DMEM) medium for 24 h. The medium was removed and fresh DMEM containing 0.5 mg/mL of Methylthiazolyldiphenyl-tetrazolium (MTT) solution was added to each well for 2 h. After that, the medium was discarded by an aspirator. The violet formazan crystals in the viable cells were dissolved in 100 μL of DMSO. The absorbance of each well was read at a wavelength of 570 nm using a microplate reader. Doxorubicin was used as a positive control with an IC_50_ value of 9.74 μM.

% cell viability = $\frac{absorbance\;of\;treated\;well}{absorbance\;of\;control\;well}$ × 100

% cytotoxicity = 100 – % cell viability

## Results and discussion

### Isolation of endophytic fungi from plant material

A total of 42 morphologically distinct endophytic fungi; 28 from tender and mature leaves, 04 from stems and 10 from bark were isolated from the collected plant specimens.

### Identification of the fungus secreting the major compound

The endophytic fungus isolated from a mature leaf of the *Bruguiera gymnorrhyza* plant was identified as *Nigrospora sphaerica*. This was found by analyzing the DNA sequence of the ITS ribosomal RNA gene analysis and the closest GeneBank accession number obtained when deposited in the GeneBank is MG171196.

### Biological activities of the crude extract

The agar disc diffusion assay results revealed that the crude ethyl acetate extract of the endophytic fungi *Nigrospora sphaerica* was active against *B. subtilis* (UBC 344) – 10 mm (diameter), *S. aureus* (ATCC 43,300) – 14 mm, methicillin resistant *S. aureus* (MRSA, ATCC 33,591) – 4 mm, *E. coli* (UBC 8161) – 13 mm, *P. aeruginosa* (ATCC 27,853) – 8 mm and the pathogenic fungus *C. albicans* (ATCC 90,028) – 3 mm. These results were found to be consistent with the isolated major compound.

### Isolation and structure elucidation of the major compound

**Figure 1.**
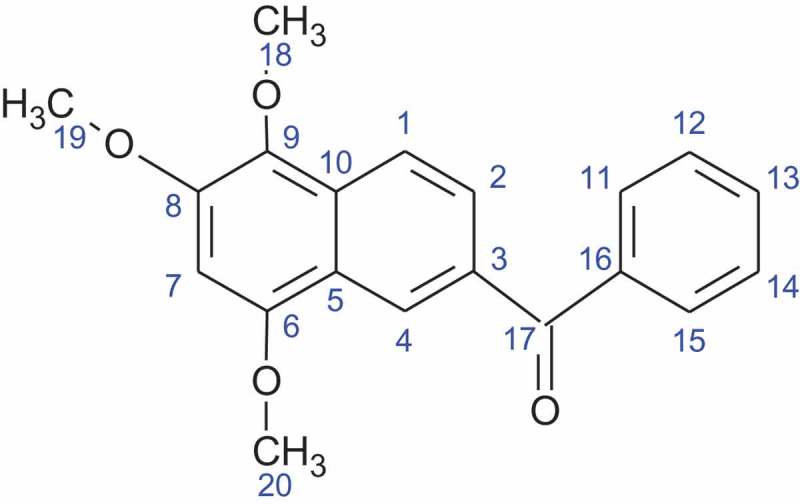
Chemical structure of Nigronapthaphenyl.

The bioassay guided fractionation of the EtOAc extract led to the isolation of the active compound (2.6 mg) as a bright orange crystalline solid eluting at a retention time of 31 minutes under the HPLC conditions (60%:40% H_2_0: MeOH) used. This compound gave a [M + Na]^+^ ion in the HR-ESI-MS at *m/z* 345.36,789 corresponding for the molecular formula of C_20_H_18_O_4_ (Figure 1). Analysis of ^1^H and ^13^C NMR data (Table 1) as well as 2D NMR (COSY, HSQC, HMBC and tROESY) spectral data (Table 3) in CDCl_3_ revealed that the structure of the active compound has not been previously reported in any literature.

The following compounds exhibit close similarities and strong resemblance to the structure of nigronapthaphenyl. Methanone, 2-naphthalenyl(3,4,5-trimethoxyphenyl)-, *O*-acetyloxime, Brunner and Petrini (2014) having three methoxy groups attached at positions C-12, C-13 and C-14 with a N-OH group attached to C-17 and secondly, methanone, (3,4-dimethoxyphenyl)(6-methoxy-2-naphthalenyl) Helvola et al. (2001) having one methoxy group at C-8 and two methoxy groups at C-12 and C-13. Methanone, (2-hydroxyphenyl)(6,7,8-trimethoxy-2-naphthalenyl) Feng and Ma (2010) has an identical carbon moiety as nigronapthaphenyl, but the three methoxy groups are attached at C-12, C-13 and C-14.

### Antimicrobial activities of nigronapthaphenyl

Nigronapthaphenyl showed strong selective antibacterial activities against Gram-positive *Bacillus subtilits* (UBC 344), *Bacillus subtilis* TISTR 088, *Bacillus cereus* TISTR 688, *S. aureus* (ATCC 43,300) and MRSA (ATCC 33,591) with MIC values of 4, 4, 2, 4 and 2 μg mL^−1^ respectively, and also towards Gram negative *E. coli* (UBC 8161) and *P. aeruginosa* (ATCC 27,853) both, showing inhibition at 2 μg mL^−1^ (Table 2). It also showed promising activity against the pathogenic fungi *C. albicans* (ATCC 90,028) *C. gloeosporioides* (UBC 3110) and *A. niger* (UBC 9214), 2, 4 and 8 μg mL^−1^ respectively. Polymixin B, amphotericin and rifamycin were used as positive controls and their respective MIC values are given in Table 4.

**Table 1.** ^13^C NMR data of Nigronapthaphenyl.

C#	13C δ (ppm)	C#	13C δ (ppm)	C#	13C δ (ppm)	C#	13C δ (ppm)
1	129.1	6	138.7	11	131.2	16	130.4
2	128.2	7	98.7	12	130.2	17	196.4
3	126.5	8	154.4	13	133.4	18	58.3
4	129.3	9	152.2	14	130.3	19	61.3
5	133.4	10	132.3	15	131.3	20	58.2

**Table 2.** ^1^H NMR data of Nigronapthaphenyl.

C#	1H δ (ppm)	C#	1H δ (ppm)	C#	1H δ (ppm)	C#	1H δ (ppm)
1	8.00	6	-	11	7.87	16	-
2	8.15	7	6.69	12	7.62	17	-
3	-	8	-	13	7.53	18	4.17
4	8.63	9	-	14	7.52	19	3.84
5	-	10	-	15	7.88	20	3.91

**Table 3.** HSQC and HMBC correlations of Nigronapthaphenyl.

C#	HSQC (ppm)	HMBC
1	8.00	2,3
2		1,3,10
3		-
4	8.63	3,5
5		-
6		-
7	6.69	6,8,9
8		-
9		-
10		-
11	7.88	12,16
12		11,13
13		12,14,15
14	7.53	13,15
15		14,16
16		-
17		-
18	4.18	6
19	3.98	8
20	4.10	9

**Table 4.** MIC values obtained for Nigronapthaphenyl and for positive controls.

	Minimum Inhibitory Concentration Values (MIC) – µg/mL									
Compound Name	*B. subtilis*	*B. subtilis* *TISTR 088*	*B. cereus TISTR* *688*	*S. aureus*	*E. coli*	MRSA	*C. albicans*	*P. aeruginosa*	*C. gloeosporioides*	*A. niger*
Nigronapthaphenyl	**4**	**4**	**2**	**4**	**2**	**2**	**2**	**2**	**4**	**8**
Polymixin B	4	1	1	-	1	-	-	1	-	-
Amphotericin	-	-	-	-	-	-	1	-	1	1
Rifamycin	-	-	-	1	-	1	-	-	-	1

### Anti-inflammatory activity of nigronapthaphenyl

This compound exhibited a value of 41% (results were expressed as percentages of IL6 relative to *E. coli*-stimulated blood, therefore, lower the number showed greater anti-inflammatory activity.) This compound showed an IC_50_ of 6.2 ± 0.5 μM.

### α-Glucosidase inhibitory activity of nigronapthaphenyl

Nigronapthaphenyl showed a significant potential α-glucosidase inhibitory activity, with an IC_50_ value of 6.9 ± 0.5 μM.

### Cytotoxicity of nigronapthaphenyl

This compound gave rise to a cell viability value of 39%, exhibiting the cytotoxicity to be 61%. This compound also showed cytotoxicity against a colon cancer cell line HCT 116 with IC_50_ values of 9.62 ± 0.5 μM.

## Conclusion

This is the first report of isolation, structure elucidation and determination of antibacterial, anti-fungal, anti-inflammatory, α glucosidase inhibitory and cytotoxicity activities of Nigronapthaphenyl, isolated from the extract of *Nigrospora sphaerica –* an endophytic fungus of mangrove plant *Bruguiera gymnorrhyza.*
